# 3-Acetyl-1-(3-methyl­phen­yl)thio­urea

**DOI:** 10.1107/S1600536812032825

**Published:** 2012-07-28

**Authors:** B. Thimme Gowda, Sabine Foro, Sharatha Kumar

**Affiliations:** a Department of Chemistry, Mangalore University, Mangalagangotri 574 199, Mangalore, India; b Institute of Materials Science, Darmstadt University of Technology, Petersenstrasse 23, D-64287 Darmstadt, Germany

## Abstract

In the crystal structure of the title compound, C_10_H_12_N_2_OS, the conformation of the two N—H bonds are *anti* to each other. The amide C=O and the C=S are are also *anti* to each other. The N—H bond adjacent to the benzene ring is *syn* to the *m*-methyl groups. The dihedral angle between the benzene ring and the side chain [mean plane of atoms C—C(O)N—C—N; maximum deviation 0.029 (2) Å] is 14.30 (7)°. There is an intramolecular N—H⋯O hydrogen bond generating an *S*(6) ring motif. In the crystal, the molecules are linked *via* N—H⋯) hydrogen bonds, forming chains propagating along [001]. The S atom is disordered and was refined using a split model [occupancy ratio 0.56 (4):0.44 (4)].

## Related literature

For studies on the effects of substituents on the structures and other aspects of *N*-(ar­yl)-amides, see: Alkan *et al.* (2011 ▶); Bhat & Gowda (2000 ▶); Bowes *et al.* (2003 ▶); Gowda *et al.* (2000 ▶); Saeed *et al.* (2010 ▶); Shahwar *et al.* (2012 ▶), of *N*-(ar­yl)-methane­sulfonamides, see: Gowda *et al.* (2007 ▶) and of *N*-chloro­aryl­sulfonamides, see: Gowda & Ramachandra (1989 ▶); Jyothi & Gowda (2004 ▶); Shetty & Gowda (2004 ▶).
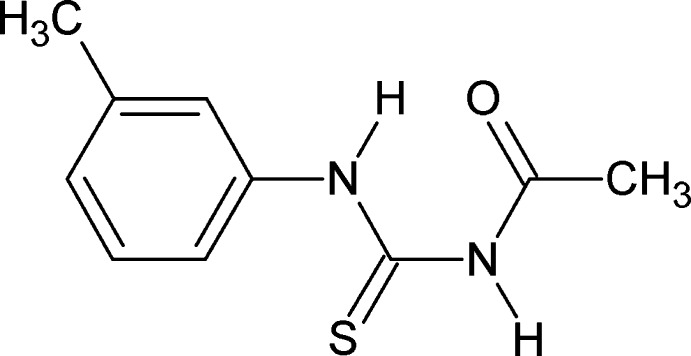


## Experimental

### 

#### Crystal data

C_10_H_12_N_2_OS
*M* *_r_* = 208.29
Monoclinic, 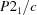

*a* = 7.6841 (9) Å
*b* = 14.943 (1) Å
*c* = 9.5358 (9) Å
β = 107.49 (1)°
*V* = 1044.32 (18) Å^3^
*Z* = 4
Mo *K*α radiation
μ = 0.28 mm^−1^
*T* = 295 K
0.48 × 0.44 × 0.24 mm

#### Data collection

Oxford Diffraction Xcalibur Sapphire CCD. diffractometer
Absorption correction: multi-scan (*CrysAlis RED*; Oxford Diffraction, 2009 ▶) *T*_min_ = 0.878, *T*_max_ = 0.936
4011 measured reflections
2137 independent reflections
1789 reflections with *I* > 2σ(*I*)
*R*_int_ = 0.011

#### Refinement

*R*[*F* ^2^ > 2σ(*F* ^2^)] = 0.035
*wR*(*F* ^2^) = 0.100
*S* = 1.06
2137 reflections
145 parameters
2 restraints
H atoms treated by a mixture of independent and constrained refinement
Δρ_max_ = 0.17 e Å^−3^
Δρ_min_ = −0.22 e Å^−3^

### 

Data collection: *CrysAlis CCD* (Oxford Diffraction, 2009 ▶); cell refinement: *CrysAlis CCD*; data reduction: *CrysAlis RED* (Oxford Diffraction, 2009 ▶); program(s) used to solve structure: *SHELXS97* (Sheldrick, 2008 ▶); program(s) used to refine structure: *SHELXL97* (Sheldrick, 2008 ▶); molecular graphics: *PLATON* (Spek, 2009 ▶); software used to prepare material for publication: *SHELXL97*.

## Supplementary Material

Crystal structure: contains datablock(s) I, global. DOI: 10.1107/S1600536812032825/rk2375sup1.cif

Structure factors: contains datablock(s) I. DOI: 10.1107/S1600536812032825/rk2375Isup2.hkl

Supplementary material file. DOI: 10.1107/S1600536812032825/rk2375Isup3.cml

Additional supplementary materials:  crystallographic information; 3D view; checkCIF report

## Figures and Tables

**Table 1:** Hydrogen-bond geometry (Å, °)

*D*—H⋯*A*	*D*—H	H⋯*A*	*D*⋯*A*	*D*—H⋯*A*
N1—H1*N*⋯O1	0.87 (1)	1.90 (2)	2.6536 (16)	144 (2)
N2—H2*N*⋯O1^i^	0.85 (1)	2.12 (1)	2.9564 (16)	166 (2)

Symmetry code: (i) 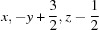.

**Fig. 1.**
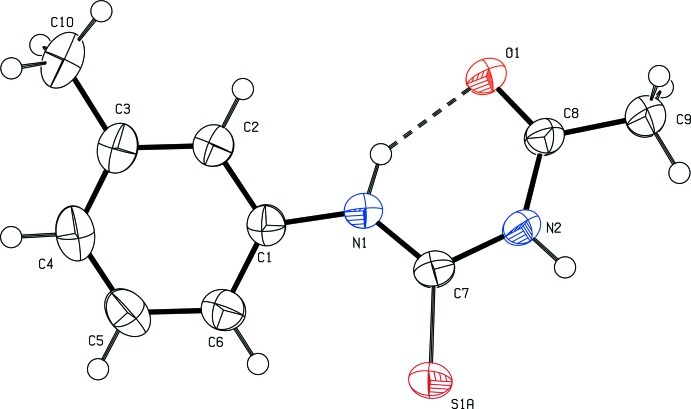
Molecular structure of the title compound, showing the atom labelling scheme. Displacement ellipsoids are drawn at the 50% probability level. H atoms are presented as small spheres of arbitrary radius. Only major moiety (S1A) are presented.

**Fig. 2.**
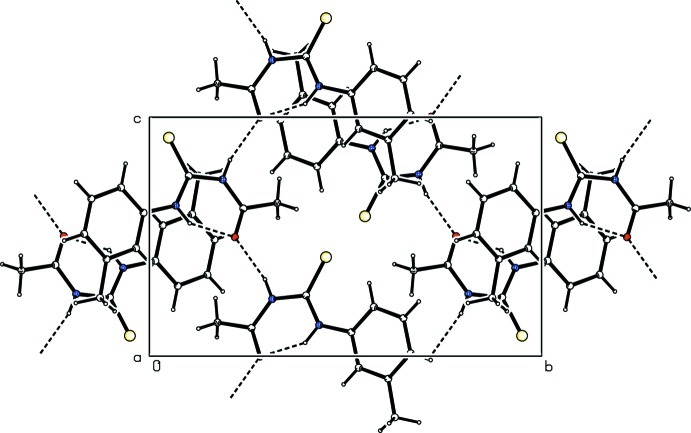
Molecular packing of the title compound with hydrogen bonding shown as dashed lines.

## supplementary crystallographic information

### Comment

Thiourea and its derivatives are known to exhibit a wide variety of biological activities. As part of our studies on the substituent effects on the structures and other aspects of *N*-(aryl)-amides (Alkan *et al.*, 2011; Bhat & Gowda, 2000; Bowes *et al.*, 2003; Gowda *et al.*, 2000; Saeed *et al.*, 2010; Shahwar *et al.*, 2012); *N*-(aryl)-methanesulfonamides (Gowda *et al.*, 2007) and *N*-chloroarylamides (Gowda & Ramachandra, 1989; Jyothi & Gowda, 2004; Shetty & Gowda, 2004), in the present work, the crystal structure of 3-acetyl-1-(3-methylphenyl)thiourea, has been determined (Fig. 1).

The conformation of the two N–H bonds are *anti* to each other. The adjacent N–H bond is *syn* to the *m*-methyl group in the benzene ring, compared to the *anti* conformation observed between the N–H bond and the *o*-methyl group in the benzene ring in 3-acetyl-1-(2-methylphenyl)thiourea, I, (Shahwar *et al.*, 2012). Furthermore, the conformation of the amide C═O and the C═S are *anti* to each other, similar to that observed in I.

The side chain is oriented itself with respect to the phenyl ring with the torsion angles of C2—C1–N1—C7 = -168.76 (14)° and C6—C1—N1—C7 = 14.71 (24)°. The dihedral angle between the phenyl ring and the side chain (N1/C7/N2/C8/O1/C9) is 14.30 (7)°.

The amide oxygen exhibits a bifurcated hydrogen bonding by showing the simultaneous intra- and intermolecular hydrogen bonding generating *S*(6) and *C*(4) motifs. In the crystal of the title compound, the molecules are linked *via* N—H···S hydrogen bonds with an *R*_2_^2^(12) motif and N—H···O hydrogen bonds with a *C*(4) motif into a layered structure (Table 1, Fig. 2).

### Experimental

3-Acetyl-1-(3-methylphenyl)thiourea was synthesized by adding a solution of acetyl chloride (0.10 mol) in acetone (30 ml) dropwise to a suspension of ammonium thiocyanate (0.10 mol) in acetone (30 ml). The reaction mixture was refluxed for 30 min. After cooling to room temperature, a solution of 3-methylaniline (0.10 mol) in acetone (10 ml) was added and refluxed for 3 h. The reaction mixture was poured into acidified cold water. The precipitated title compound was recrystallized to constant melting point from acetonitrile. The purity of the compound was checked and characterized by its infrared spectrum. The characteristic absorptions observed are 3163.7 cm^-1^, 1690.0 cm^-1^, 1269.5 cm^-1^ and 693.3 cm^-1^ for the stretching bands of N-H, C═O, C-N and C═S, respectively.

Prism like yellow single crystals used in X-ray diffraction studies were grown in acetonitrile solution by slow evaporation of the solvent at room temperature.

### Refinement

H atoms bonded to C were positioned with idealized geometry using a riding model with the aromatic C—H = 0.93Å, methyl C—H = 0.96Å. The amino H atoms were freely refined with the N–H distances restrained to 0.86 (2)Å. All H atoms were refined with isotropic displacement parameters set at 1.2 *U*_eq_(C-aromatic, N) and 1.5 *U*_eq_ (*C*-methyl) of the parent atom.

The S atom is disordered and was refined using a split model. The corresponding s.o.f.'s were refined so that their sum was unity: 0.56 (4) and 0.44.

### Crystal data

**Table:**

C_10_H_12_N_2_OS	*F*(000) = 440
*M**_r_* = 208.29	*D*_x_ = 1.325 Mg m^−^^3^
Monoclinic, *P*2_1_/*c*	Mo *K*α radiation, λ = 0.71073 Å
Hall symbol: -P 2ybc	Cell parameters from 1948 reflections
*a* = 7.6841 (9) Å	θ = 2.6–27.9°
*b* = 14.943 (1) Å	µ = 0.28 mm^−^^1^
*c* = 9.5358 (9) Å	*T* = 295 K
β = 107.49 (1)°	Prism, yellow
*V* = 1044.32 (18) Å^3^	0.48 × 0.44 × 0.24 mm
*Z* = 4	

### Data collection

**Table:**

Oxford Diffraction Xcalibur Sapphire CCD. diffractometer	2137 independent reflections
Radiation source: fine-focus sealed tube	1789 reflections with *I* > 2σ(*I*)
Graphite monochromator	*R*_int_ = 0.011
Rotation method data acquisition using ω and φ scans	θ_max_ = 26.4°, θ_min_ = 2.6°
Absorption correction: multi-scan (*CrysAlis RED*; Oxford Diffraction, 2009)	*h* = −9→8
*T*_min_ = 0.878, *T*_max_ = 0.936	*k* = −13→18
4011 measured reflections	*l* = −9→11

### Refinement

**Table:**

Refinement on *F*^2^	Primary atom site location: structure-invariant direct methods
Least-squares matrix: full	Secondary atom site location: difference Fourier map
*R*[*F*^2^ > 2σ(*F*^2^)] = 0.035	Hydrogen site location: inferred from neighbouring sites
*wR*(*F*^2^) = 0.100	H atoms treated by a mixture of independent and constrained refinement
*S* = 1.06	*w* = 1/[σ^2^(*F*_o_^2^) + (0.0535*P*)^2^ + 0.2352*P*] where *P* = (*F*_o_^2^ + 2*F*_c_^2^)/3
2137 reflections	(Δ/σ)_max_ = 0.001
145 parameters	Δρ_max_ = 0.17 e Å^−^^3^
2 restraints	Δρ_min_ = −0.22 e Å^−^^3^

### Special details

**Table:**

Experimental. *CrysAlis RED* (Oxford Diffraction, 2009) Empirical absorption correction using spherical harmonics, implemented in SCALE3 ABSPACK scaling algorithm.
Geometry. All s.u.'s (except the s.u. in the dihedral angle between two l.s. planes) are estimated using the full covariance matrix. The cell s.u.'s are taken into account individually in the estimation of s.u.'s in distances, angles and torsion angles; correlations between s.u.'s in cell parameters are only used when they are defined by crystal symmetry. An approximate (isotropic) treatment of cell s.u.'s is used for estimating s.u.'s involving l.s. planes.
Refinement. Refinement of *F*^2^ against ALL reflections. The weighted *R*-factor *wR* and goodness of fit *S* are based on *F*^2^, conventional *R*-factors *R* are based on *F*, with *F* set to zero for negative *F*^2^. The threshold expression of *F*^2^ > σ(*F*^2^) is used only for calculating *R*-factors(gt) *etc*. and is not relevant to the choice of reflections for refinement. *R*-factors based on *F*^2^ are statistically about twice as large as those based on *F*, and *R*-factors based on ALL data will be even larger.

### Fractional atomic coordinates and isotropic or equivalent isotropic displacement parameters (Å^2^)

**Table:**

	*x*	*y*	*z*	*U*_iso_*/*U*_eq_	Occ. (<1)
C1	0.20722 (18)	1.01499 (9)	0.39974 (15)	0.0337 (3)	
C2	0.23877 (19)	1.03395 (10)	0.54780 (17)	0.0364 (3)	
H2	0.3113	0.9953	0.6178	0.044*	
C3	0.1649 (2)	1.10903 (10)	0.59419 (18)	0.0413 (4)	
C4	0.0590 (2)	1.16622 (11)	0.4881 (2)	0.0494 (4)	
H4	0.0085	1.2171	0.5163	0.059*	
C5	0.0282 (2)	1.14793 (11)	0.3408 (2)	0.0506 (4)	
H5	−0.0429	1.1871	0.2710	0.061*	
C6	0.1008 (2)	1.07243 (10)	0.29439 (18)	0.0428 (4)	
H6	0.0785	1.0607	0.1948	0.051*	
C7	0.30263 (18)	0.89953 (9)	0.24567 (15)	0.0334 (3)	
C8	0.4388 (2)	0.76042 (10)	0.38195 (15)	0.0361 (3)	
C9	0.5236 (3)	0.67388 (11)	0.35913 (18)	0.0520 (4)	
H9A	0.6484	0.6724	0.4198	0.078*	
H9B	0.5190	0.6685	0.2577	0.078*	
H9C	0.4580	0.6251	0.3851	0.078*	
C10	0.1971 (2)	1.12586 (13)	0.7555 (2)	0.0539 (4)	
H10A	0.2948	1.0885	0.8117	0.081*	
H10B	0.0880	1.1123	0.7807	0.081*	
H10C	0.2289	1.1875	0.7772	0.081*	
N1	0.28202 (17)	0.93305 (8)	0.36952 (13)	0.0354 (3)	
H1N	0.323 (2)	0.8973 (10)	0.4440 (16)	0.042*	
N2	0.37949 (17)	0.81347 (8)	0.26055 (13)	0.0344 (3)	
H2N	0.391 (2)	0.7951 (11)	0.1792 (16)	0.041*	
O1	0.42351 (17)	0.78202 (7)	0.50180 (11)	0.0483 (3)	
S1A	0.2654 (12)	0.9501 (3)	0.0852 (7)	0.0498 (10)	0.56 (4)
S1B	0.234 (2)	0.9416 (8)	0.0776 (9)	0.0656 (16)	0.44 (4)

### Atomic displacement parameters (Å^2^)

**Table:**

	*U*^11^	*U*^22^	*U*^33^	*U*^12^	*U*^13^	*U*^23^
C1	0.0337 (7)	0.0314 (7)	0.0377 (8)	−0.0023 (6)	0.0131 (6)	−0.0005 (6)
C2	0.0353 (7)	0.0366 (8)	0.0373 (7)	−0.0019 (6)	0.0106 (6)	−0.0014 (6)
C3	0.0392 (8)	0.0389 (8)	0.0495 (9)	−0.0076 (6)	0.0191 (7)	−0.0089 (7)
C4	0.0506 (9)	0.0353 (8)	0.0676 (11)	0.0024 (7)	0.0256 (8)	−0.0049 (8)
C5	0.0517 (9)	0.0398 (9)	0.0612 (11)	0.0092 (7)	0.0182 (8)	0.0120 (8)
C6	0.0461 (8)	0.0421 (8)	0.0402 (8)	0.0039 (7)	0.0129 (7)	0.0052 (7)
C7	0.0344 (7)	0.0363 (7)	0.0299 (7)	−0.0020 (6)	0.0103 (5)	0.0014 (6)
C8	0.0471 (8)	0.0337 (7)	0.0297 (7)	−0.0007 (6)	0.0150 (6)	0.0016 (6)
C9	0.0795 (12)	0.0421 (9)	0.0397 (9)	0.0143 (8)	0.0256 (8)	0.0051 (7)
C10	0.0549 (10)	0.0569 (11)	0.0547 (10)	−0.0057 (8)	0.0238 (8)	−0.0196 (8)
N1	0.0451 (7)	0.0337 (6)	0.0273 (6)	0.0047 (5)	0.0108 (5)	0.0027 (5)
N2	0.0456 (7)	0.0344 (6)	0.0254 (6)	0.0008 (5)	0.0140 (5)	−0.0008 (5)
O1	0.0791 (8)	0.0417 (6)	0.0292 (6)	0.0119 (5)	0.0242 (5)	0.0047 (4)
S1A	0.078 (2)	0.043 (2)	0.0358 (14)	0.0165 (11)	0.0274 (16)	0.0155 (6)
S1B	0.076 (3)	0.091 (4)	0.0285 (11)	0.031 (2)	0.0134 (15)	0.0141 (17)

### Geometric parameters (Å, º)

**Table:**

C1—C6	1.386 (2)	C7—S1A	1.653 (5)
C1—C2	1.388 (2)	C7—S1B	1.654 (7)
C1—N1	1.4186 (18)	C8—O1	1.2268 (17)
C2—C3	1.388 (2)	C8—N2	1.3636 (18)
C2—H2	0.9300	C8—C9	1.493 (2)
C3—C4	1.386 (2)	C9—H9A	0.9600
C3—C10	1.504 (2)	C9—H9B	0.9600
C4—C5	1.379 (3)	C9—H9C	0.9600
C4—H4	0.9300	C10—H10A	0.9600
C5—C6	1.389 (2)	C10—H10B	0.9600
C5—H5	0.9300	C10—H10C	0.9600
C6—H6	0.9300	N1—H1N	0.868 (13)
C7—N1	1.3354 (18)	N2—H2N	0.853 (14)
C7—N2	1.4044 (19)		
			
C6—C1—C2	119.71 (14)	S1A—C7—S1B	9.2 (7)
C6—C1—N1	125.04 (13)	O1—C8—N2	122.46 (13)
C2—C1—N1	115.17 (13)	O1—C8—C9	122.14 (13)
C3—C2—C1	121.73 (14)	N2—C8—C9	115.40 (13)
C3—C2—H2	119.1	C8—C9—H9A	109.5
C1—C2—H2	119.1	C8—C9—H9B	109.5
C4—C3—C2	118.18 (15)	H9A—C9—H9B	109.5
C4—C3—C10	121.56 (15)	C8—C9—H9C	109.5
C2—C3—C10	120.25 (15)	H9A—C9—H9C	109.5
C5—C4—C3	120.29 (15)	H9B—C9—H9C	109.5
C5—C4—H4	119.9	C3—C10—H10A	109.5
C3—C4—H4	119.9	C3—C10—H10B	109.5
C4—C5—C6	121.54 (16)	H10A—C10—H10B	109.5
C4—C5—H5	119.2	C3—C10—H10C	109.5
C6—C5—H5	119.2	H10A—C10—H10C	109.5
C1—C6—C5	118.55 (15)	H10B—C10—H10C	109.5
C1—C6—H6	120.7	C7—N1—C1	131.79 (12)
C5—C6—H6	120.7	C7—N1—H1N	112.6 (11)
N1—C7—N2	114.34 (12)	C1—N1—H1N	115.7 (11)
N1—C7—S1A	127.9 (2)	C8—N2—C7	129.89 (12)
N2—C7—S1A	117.58 (19)	C8—N2—H2N	119.0 (11)
N1—C7—S1B	128.8 (3)	C7—N2—H2N	111.0 (11)
N2—C7—S1B	116.6 (3)		
			
C6—C1—C2—C3	0.6 (2)	N2—C7—N1—C1	−178.23 (13)
N1—C1—C2—C3	−176.12 (12)	S1A—C7—N1—C1	7.4 (5)
C1—C2—C3—C4	−0.8 (2)	S1B—C7—N1—C1	−4.2 (10)
C1—C2—C3—C10	178.03 (14)	C6—C1—N1—C7	14.7 (2)
C2—C3—C4—C5	0.3 (2)	C2—C1—N1—C7	−168.76 (14)
C10—C3—C4—C5	−178.41 (16)	O1—C8—N2—C7	3.5 (2)
C3—C4—C5—C6	0.2 (3)	C9—C8—N2—C7	−176.63 (14)
C2—C1—C6—C5	−0.1 (2)	N1—C7—N2—C8	−1.4 (2)
N1—C1—C6—C5	176.32 (14)	S1A—C7—N2—C8	173.5 (4)
C4—C5—C6—C1	−0.3 (2)	S1B—C7—N2—C8	−176.3 (8)

### Hydrogen-bond geometry (Å, º)

**Table:**

*D*—H···*A*	*D*—H	H···*A*	*D*···*A*	*D*—H···*A*
N1—H1*N*···O1	0.87 (1)	1.90 (2)	2.6536 (16)	144 (2)
N2—H2*N*···O1^i^	0.85 (1)	2.12 (1)	2.9564 (16)	166 (2)

Symmetry code: (i) *x*, −*y*+3/2, *z*−1/2.
